# Supplementary material for: Discovery and application of insertion-deletion (INDEL) polymorphisms for QTL mapping of early life-history traits in Atlantic salmon
Source: BMC Genomics. 2010 Mar 8;11:156. doi: 10.1186/1471-2164-11-156 (PMC2838853; doi:10.1186/1471-2164-11-156)
Supplement: Additional file 2 — Information on developed 76 locus single-run INDEL panel in Atlantic salmon. Information on fluorescence labeling, primer concentrations, PCR pooling and links to alignments, INDEL motifs and GENESCAN (Burge and Karlin 1997) predictions of genes/exons are available in html format. [file 1471-2164-11-156-S2.ZIP › Additionalfile2/snpsummary4868.html]

```
Cluster 1182 Contig 1

prev  Summary    Contig List  next
```

Size of Consensus sequence = 1240

Number of sequences = 26

Minimum redundancy = 6

Key

A gi|117437801|gb|EG770024.1|EG770024 EST\_ssal\_evd\_45805 ssalevd thymus Salmo salar cDNA Salmo salar cDNA clone ssal\_evd\_561\_200\_rev 5', mRNA sequence  
B gi|117447411|gb|EG779630.1|EG779630 EST\_ssal\_evd\_36451 ssalevd thymus Salmo salar cDNA Salmo salar cDNA clone ssal\_evd\_548\_252\_rev 5', mRNA sequence  
C gi|117447119|gb|EG779338.1|EG779338 EST\_ssal\_evd\_36187 ssalevd thymus Salmo salar cDNA Salmo salar cDNA clone ssal\_evd\_548\_118\_rev 5', mRNA sequence  
D gi|117447120|gb|EG779339.1|EG779339 EST\_ssal\_evd\_36188 ssalevd thymus Salmo salar cDNA Salmo salar cDNA clone ssal\_evd\_548\_118\_fwd 3', mRNA sequence  
E gi|117480426|gb|EG812643.1|EG812643 EST\_ssal\_evd\_18309 ssalevd thymus Salmo salar cDNA Salmo salar cDNA clone ssal\_evd\_523\_180\_rev 5', mRNA sequence  
F gi|117480439|gb|EG812656.1|EG812656 EST\_ssal\_evd\_18310 ssalevd thymus Salmo salar cDNA Salmo salar cDNA clone ssal\_evd\_523\_180\_fwd 3', mRNA sequence  
G gi|50135670|gb|CO470159.1|CO470159 SGP170089 Atlantic salmon Eye cDNA library Salmo salar cDNA clone OY4-1589 5', mRNA sequence  
H gi|117457941|gb|EG790160.1|EG790160 EST\_ssal\_evd\_10464 ssalevd thymus Salmo salar cDNA Salmo salar cDNA clone ssal\_evd\_512\_301\_rev 5', mRNA sequence  
I gi|117479934|gb|EG812151.1|EG812151 EST\_ssal\_evd\_32256 ssalevd thymus Salmo salar cDNA Salmo salar cDNA clone ssal\_evd\_542\_315\_rev 5', mRNA sequence  
J gi|117447412|gb|EG779631.1|EG779631 EST\_ssal\_evd\_36452 ssalevd thymus Salmo salar cDNA Salmo salar cDNA clone ssal\_evd\_548\_252\_fwd 3', mRNA sequence  
K gi|117475883|gb|EG808102.1|EG808102 EST\_ssal\_evd\_28611 ssalevd thymus Salmo salar cDNA Salmo salar cDNA clone ssal\_evd\_537\_177\_rev 5', mRNA sequence  
L gi|83641202|gb|DW007792.1|DW007792 SGP151699 Atlantic salmon Red muscle cDNA library Salmo salar cDNA clone RM6-0319 5', mRNA sequence  
M gi|117437802|gb|EG770025.1|EG770025 EST\_ssal\_evd\_45806 ssalevd thymus Salmo salar cDNA Salmo salar cDNA clone ssal\_evd\_561\_200\_fwd 3', mRNA sequence  
N gi|117450686|gb|EG782905.1|EG782905 EST\_ssal\_evd\_39398 ssalevd thymus Salmo salar cDNA Salmo salar cDNA clone ssal\_evd\_552\_279\_rev 5', mRNA sequence  
O gi|117468356|gb|EG800575.1|EG800575 EST\_ssal\_evd\_57300 ssalevd thymus Salmo salar cDNA Salmo salar cDNA clone ssal\_evd\_577\_107\_rev 5', mRNA sequence  
P gi|117457940|gb|EG790159.1|EG790159 EST\_ssal\_evd\_10463 ssalevd thymus Salmo salar cDNA Salmo salar cDNA clone ssal\_evd\_512\_301\_fwd 3', mRNA sequence  
Q gi|117444462|gb|EG776685.1|EG776685 EST\_ssal\_evd\_51801 ssalevd thymus Salmo salar cDNA Salmo salar cDNA clone ssal\_evd\_569\_308\_rev 5', mRNA sequence  
R gi|117444463|gb|EG776686.1|EG776686 EST\_ssal\_evd\_51802 ssalevd thymus Salmo salar cDNA Salmo salar cDNA clone ssal\_evd\_569\_308\_fwd 3', mRNA sequence  
S gi|117468357|gb|EG800576.1|EG800576 EST\_ssal\_evd\_57301 ssalevd thymus Salmo salar cDNA Salmo salar cDNA clone ssal\_evd\_577\_107\_fwd 3', mRNA sequence  
T gi|45322318|gb|CK892585.1|CK892585 SGP152635 Atlantic salmon Skin cDNA library Salmo salar cDNA clone HU5-0175 5', mRNA sequence  
U gi|117450687|gb|EG782906.1|EG782906 EST\_ssal\_evd\_39399 ssalevd thymus Salmo salar cDNA Salmo salar cDNA clone ssal\_evd\_552\_279\_fwd 3', mRNA sequence  
V gi|117457559|gb|EG789778.1|EG789778 EST\_ssal\_evd\_53515 ssalevd thymus Salmo salar cDNA Salmo salar cDNA clone ssal\_evd\_572\_048\_rev 5', mRNA sequence  
W gi|117475884|gb|EG808103.1|EG808103 EST\_ssal\_evd\_28612 ssalevd thymus Salmo salar cDNA Salmo salar cDNA clone ssal\_evd\_537\_177\_fwd 3', mRNA sequence  
X gi|117488592|gb|EG820809.1|EG820809 EST\_ssal\_evd\_16716 ssalevd thymus Salmo salar cDNA Salmo salar cDNA clone ssal\_evd\_521\_131\_fwd 3', mRNA sequence  
Y gi|50135764|gb|CO470206.1|CO470206 SGP170137 Atlantic salmon Eye cDNA library Salmo salar cDNA clone OY4-1643 5', mRNA sequence  
Z gi|117457548|gb|EG789767.1|EG789767 EST\_ssal\_evd\_53514 ssalevd thymus Salmo salar cDNA Salmo salar cDNA clone ssal\_evd\_572\_048\_fwd 3', mRNA sequence

9 SNPs detected

A B C D E F G H I J K L M N O P Q R S T U V W X Y Z  cosegregation weighted

246 A A A A A A - A A A A - A A - A - - - . A . . . . .   9/9 76.92
247 A A A A A A - A A A A - A A - A - - - . A . . . . .   9/9 76.92
248 A A A A A A - A A A A - A A - A - - - . A . . . . .   9/9 76.92
249 C C C C C C - C C C C - C C - C - - - . C . . . . .   9/9 76.92
601 . . . . . . . G G G G T G G T G T T T T G . G G . .   9/9 61.54
632 . . . . . . . . A A A - A A - A - - - - A A A A . A   9/9 65.38
633 . . . . . . . . T T T - T T - T - - - - T T T T . T   9/9 65.38
634 . . . . . . . . A A A - A A - A - - - - A A A A . A   9/9 65.38
635 . . . . . . . . T T T - T T - T - - - - T T T T . T   9/9 65.38
